# Supplementary material for: Content-rich biological network constructed by mining PubMed abstracts
Source: BMC Bioinformatics. 2004 Oct 8;5:147. doi: 10.1186/1471-2105-5-147 (PMC528731; doi:10.1186/1471-2105-5-147)
Supplement: Additional File 5 — The original Chilibot query results of the term "long-term potentiation (LTP)" and 22 other terms, limiting the latest references analyzed to the years 1990, 1995, 2000, and 2004. [file 1471-2105-5-147-S5.bz2 › chilibotAdditionalFile5/ltp2004/html/CREB_PI-3K.html]

 


 **CREB** and **PI-3K** 
  
Found 28 abstracts in PubMed,  **28 abstracts were retrieved and analyzed**.  


---

 Search Google  |
 PDF files only 
|  EDU domain only 

---

**Interactive relationship** (e.g. stimulation, inhibition, etc)

- We propose that NMDA receptor stimulation can activate Erk1 2 and Akt signalling pathways in a PI 3 kinase  [ **PI-3K** ]  dependent manner which may target  **CREB**  in the nucleus.  Ref: 11902114 J Neurochem, 2002
- Tetanus and forskolin induced activation of mitogen activated protein kinase MAPK was blocked by PI 3 kinase  [ **PI-3K** ]  inhibitors, which also inhibited cAMP response element binding protein  **CREB**  phosphorylation.  Ref: 11567621 Neuron, 2001
- Likewise, PI 3 kinase  [ **PI-3K** ]  inhibitor blocked fear training induced cAMP response element binding protein  **CREB**  phosphorylation as well as extinction training induced decrease in  **CREB**  phosphorylation, the latter of which was associated with calcineurin expression and could be reversed by a specific calcineurin inhibitor.  Ref: 12967993 J Neurosci, 2003
- However, AMPA receptor stimulation led to a calcium dependent phosphorylation of the nuclear transcription factor  **CREB** , which could be prevented by inhibitors of MEK or PI 3 kinase  [ **PI-3K** ] .  Ref: 10407026 J Neurosci, 1999
- Further, PDGF, PI 3 kinase  [ **PI-3K** ] , and Rac1 each activated the cyclin D 1 promoter at the cyclic adenosine monophosphate response element binding protein  **CREB**  activating transcription factor ATF 2 binding site, as evidenced by expression of a  **CREB**  ATF 2 reporter plasmid.  Ref: 11017907 Am J Respir Cell Mol Biol, 2000
- These interactions included a central role of tyrosine phosphorylation for stimulation of PKA  **CREB** , MAPK and PI3 kinase  [ **PI-3K** ]  PKB, inhibition of PKA  **CREB**  by the MAPK pathway at the level of MAPK kinase 1 or downstream, activation of MAPK signaling by PI3 kinase  [ **PI-3K** ]  and PKA at the level of extracellular signal regulated kinase 1 2 or upstream, and activation of PKB by MAPK and PKA signaling at the level of PKB or upstream.  Ref: 12176662 J Endocrinol, 2002
- Constitutively active and dominant negative regulators of the Ras and PI 3 kinase  [ **PI-3K** ]  pathways confirmed the contribution of these pathways for  **CREB**  regulation by IGF I.  Ref: 9915817 J Biol Chem, 1999

**Parallel relationship** (e.g. studied together, co-existance, homology, etc.)

- Downstream targets of PI 3 kinase  [ **PI-3K** ] , such as Akt and the transcription factor cAMP response element binding protein  **CREB** , are activated by insulin and NT 3 and regulate sensory neuron gene expression.  Ref: 14641647 J Peripher Nerv Syst, 2003
- Finally, PI 3 kinase  [ **PI-3K** ]  and Rac1 induced  **CREB**  ATF 2 transactivation were each inhibited by catalase.  Ref: 11017907 Am J Respir Cell Mol Biol, 2000
- Moreover, TRAIL increased  **CREB**  phosphorylation and phospho  **CREB**  DNA binding activity in a phosphatidylinositol 3 kinase PI 3K  [ **PI-3K** ]  Akt dependent manner.  Ref: 12807432 J Neurochem< MedlineTA>J Neurochem, 2000
- Inhibition of MAPK kinase with PD98059, PI 3 kinase  [ **PI-3K** ]  with wortmannin, and p38 MAPK with SB203580 blocked IGF I mediated phosphorylation and transcriptional activation of  **CREB**  by 30 50% p <.  Ref: 9915817 J Biol Chem, 1999
- These data indicate that regulation of Bcl 2 expression by IGF I involves a signaling cascade mediated by PI 3 kinase  [ **PI-3K** ]  PDK1 Akt  **CREB** .  Ref: 10753867 J Biol Chem, 2000
